# Scalable Unimodal and Multimodal Deep Learning for Multi-Label Chest Disease Detection: A Comparative Analysis

**DOI:** 10.3390/diagnostics16050734

**Published:** 2026-03-01

**Authors:** Diğdem Orhan, Murat Ucan, Reda Alhajj, Mehmet Kaya

**Affiliations:** 1 Department of Computer Engineering, Firat University, Elazig 23119, Turkey; dorhan23@gmail.com; 2 Department of Computer Technologies, Dicle University, Diyarbakir 21200, Turkey; murat.ucan@dicle.edu.tr; 3 Department of Computer Science, University of Calgary, Calgary, AB T2N 1N4, Canada; 4 Department of Computer Engineering, Istanbul Medipol University, Istanbul 34810, Turkey; 5 Department of Health Informatics, University of Southern Denmark, 5230 Odense, Denmark

**Keywords:** deep learning, chest diseases, multimodal fusion, multi-label classification

## Abstract

**Background/Objectives**: Early and accurate diagnosis of chest diseases is a critical challenge in clinical practice, particularly in scenarios where multiple pathologies may coexist. While deep learning-based medical image analysis has shown promising results, most existing studies rely on unimodal data and fixed-scale datasets, limiting their generalizability and clinical relevance. In this study, we present a comprehensive comparative analysis of unimodal and multimodal deep learning models for multi-label chest disease classification using chest X-ray images and associated clinical metadata. **Methods**: A total of twelve models were developed based on three widely used convolutional neural network architectures—ResNet50, EfficientNetB3, and DenseNet121—under both unimodal (image-only) and multimodal (image + clinical data) configurations. To systematically investigate the impact of data scale, experiments were conducted on two distinct versions: the Random Sample of NIH Chest X-ray Dataset and the NIH Chest X-ray Dataset, containing 5606 and 121,120 samples, respectively. Model performance was evaluated using label-based Area Under the Receiver Operating Characteristic Curve (AUROC) metrics. **Results**: Experimental results demonstrate that multimodal fusion consistently outperforms unimodal approaches across all architectures and data scales, with more pronounced improvements observed in large-scale settings. Furthermore, increasing data volume leads to improved generalization and reduced performance variance, particularly for rare pathologies. **Conclusions**: These findings highlight the effectiveness of multimodal, multi-label learning in enhancing diagnostic accuracy and support the development of robust clinical decision support systems for chest disease assessment.

## 1. Introduction

The diagnosis of chest diseases, the monitoring of their progression, and the process of establishing a diagnosis have undergone a paradigm shift in recent years with the integration of artificial intelligence into healthcare systems. Deep learning, machine learning, and various subfields of artificial intelligence hold great promise in understanding and processing large and complex biomedical datasets [1,2]. The widespread use of large-scale annotated datasets and the development of advanced computational techniques have enabled intelligent AI support systems to recognize and detect patterns associated with various diseases [3].

In this context, the recent popularity of deep learning-based approaches has made them a frequently preferred method for providing solutions to challenges in the biomedical field, particularly in medical image automatic segmentation [4]. One such approach, single-modal modeling [5], operates using a single type of data, such as medical images or clinical text data. However, this also brings with it some disadvantages, such as limitations and applicability constraints. When the complexity of the medical image or clinical data cannot be fully resolved, some important signs and findings may be overlooked, and anomalies may not be detected [6,7]. In this case, it can lead to misinterpretation and misleading results. These shortcomings highlight the need to use multimodal systems that combine different types of sources.

In contrast, multimodal systems take an approach that integrates data from multiple sources and brings this information together. This combination allows for a more comprehensive treatment of the therapeutic problem at hand and provides a more comprehensive description. This enables clinical artificial intelligence decision support systems used in the medical field to capture complementary features across multiple modalities [8].

It is used to process and scan large-scale medical data, particularly in clinical care, and to effectively manage disease diagnosis and assessment processes. Deep learning-based multimodal medical data fusion can efficiently extract and integrate the distinctive features of various modalities. This makes it possible to improve clinical practice in diagnosis and medical evaluation and to perform real-time monitoring, treatment planning, and quantitative analysis [9].

In biomedical research, when researchers compared the algorithms they developed for classifying chest diseases using deep learning models with the performance of human experts, they demonstrated significant gains in diagnosis and healthcare [10,11]. However, existing studies differ substantially in terms of task formulation (binary vs. multi-label classification), modality structure (unimodal vs. multimodal), and evaluation strategies, which makes direct performance comparison non-trivial and sometimes misleading when metrics such as accuracy and AUC are reported without normalization or task-level context [12]. Thematic grouping of prior work. Existing studies can be broadly grouped into three main categories: (i) Unimodal multi-label models, focusing only on imaging or signal data; (ii) Multimodal binary classification models, integrating multiple data sources but restricting the task to binary decisions; (iii) Multimodal multi-label models, aiming to model co-existing pathologies across modalities.

In the multimodal binary classification group, Xu et al. proposed the Neighbor-Assisted Multimodal Integration Network (NAMAN) model, combining image–text integration and nearest-neighbor search, and reported an AUC of 0.6703 on the MIMIC-CXR and OpenI datasets [13]. They reported an AUC of 0.6703 on the MIMIC-CXR and OpenI datasets. Despite being multimodal, this comparatively low AUC suggests weak baseline performance rather than strong multimodal reasoning and limited discriminative capacity. Similarly to this, Shimbre et al. created the ChestXFusionNet model, which achieved 92% accuracy in a binary classification setting by combining clinical and chest X-ray data using 2D CNN and 1D deep learning architectures [14]. However, binary task formulation reduces real-world clinical relevance by simplifying the clinical problem and limiting the model’s capacity to represent co-occurring diseases.

Han et al. employed a multi-labeled DenseNet model on 12-lead ECG data in the unimodal or weakly multimodal multi-label category, combining deep features with manually created features. They reported an accuracy of 0.602 on the validation dataset [15], indicating limited generalization capacity in complex multi-label settings. With AUC values of 0.8309 and 0.8265, Uçan et al. presented an image-based deep learning approach for multi-class automatic diagnosis of 14 chest diseases using EfficientNet architectures and attention mechanisms [16]. These unimodal methods lack semantic and clinical context integration, despite being successful in image-based classification.

In the multimodal multi-label group, Jin et al. integrated ConvNeXt-based visual features with BioBERT semantic vectors using a dual-weighted metric loss and obtained an AUC of 0.826 [17], while Townsell et al. combined pre-trained image networks with patient-image metadata and obtained an AUC of 0.84 [18]. Using pre-trained text, image, and cross-modal encoders on the MIMIC-CXR dataset, Yang et al.’s MCX-Net produced an AUC of 0.816 [19]. A multimodal fusion deep neural network (MFDNN) for lung cancer detection was proposed by Sangeetha et al., who reported accuracy values above 86% in various evaluation settings [20]. While these studies show the potential of multimodal learning, the deep semantic alignment between image and clinical/textual information is limited because most of them rely on relatively shallow fusion strategies and limited cross-modal interaction.

The literature generally demonstrates that although high accuracy and AUC values are frequently reported, many results are not directly comparable because of variations in task definitions, modality structures, and evaluation protocols. Furthermore, a number of studies that assert multi-label learning still operate under binary or simplified clinical assumptions, failing to reflect real-world scenarios where multiple chest diseases coexist, and current multimodal approaches often fail to model deep semantic relationships between modalities [21].

However, the impact of data volume on single-modal and multi-modal learning processes has not been comprehensively investigated, and the vast majority of studies in the literature have been evaluated using a single data scale. In particular, there are very few studies that use the same architecture and preparation processes to compare single-modal and multi-modal models across different data scales. Therefore, it is difficult to determine how modality integration and data volume affect model performance. When examining the relationships between modalities, the ability to determine which modality’s information is more effective is limited, and it becomes difficult to understand which modality is more dominant and decisive for a particular disease. This situation creates a disadvantage in terms of interpreting and explaining the models. For this reason, it is necessary to develop clinical decision support systems using artificial intelligence in the biomedical field and to fill these gaps in the literature.

This study proposes models that integrate multi-label single-modality and multi-label multi-modality chest X-ray images and the associated clinical data related to these X-rays. Based on the NIH Chest X-Ray dataset, these models were compared using different CNN models (ResNet50, EfficientNetB3, DenseNet121) for single-modality and multi-modality. To examine the effect of different data sizes on pathologies, two distinct versions, the Random Sample of NIH Chest X-ray Dataset and the NIH Chest X-ray Dataset, consisting of 5606 and 121,120 images, respectively, were considered. The single-modality and multi-modality modalities in both sample groups were trained using the ResNet50, EfficientNetB3, and DenseNet121 architectures. Loss function and label-based AUC score were used as performance metrics. In this study, where a total of 12 models were constructed, including single-modal multi-label and multi-modal multi-label models, it was found that multi-modal multi-label models showed better results on performance parameters in both low and high data groups (especially high). To this end, looking at the main contributions of the study:

- Perform a comparative analysis by systematically processing different CNN architectures (ResNet50, EfficientNetB3, DenseNet121) in both single-modal and multi-modal architectures.
- Observe how different models are affected in a multi-label classification task on clusters of different sizes within the same dataset, without the constraint of binary classification.
- Demonstrate that performance issues are overcome with minimum loss values and pathological label-based AUC scores, minimizing gaps in the literature such as limited modality interaction, interpretability, and explainability.
- Make a significant contribution to the applicability of multi-label multi-modal deep learning in the medical field, emphasizing the importance of innovative models and clinical artificial intelligence decision support systems.
- Comprehensively evaluate the relationship between data quantity and modality integration by examining the generalization behavior of single-modal and multi-modal models across various data scales under the same architecture and training procedures.

## 2. Materials and Methods

### 2.1. Dataset and Preprocessing Steps

This study utilized the National Institutes of Health Chest X-ray (NIH Chest X-ray14) dataset [22]. This dataset contains 14 different diseases with chest X-ray data matched with labels. In the relevant study, two different versions of the dataset available on the Kaggle platform were selected: the NIH Chest X-ray Dataset, consisting of 121,120 data points [22], and the Random Sample of NIH Chest X-ray Dataset, consisting of 5606 data points [23]. The two groups, containing different numbers of examples, do not differ in terms of the content of clinical data and medical image data, but only in the number of examples belonging to pathological disease classes. Table 1 provides an example of the file containing clinical data.

As seen in the sample data presented in Table 1, the clinical data in the dataset consists of 11 columns. The Image Index column contains the image data matched with the X-ray data. Finding Labels contains disease columns and, as shown in the table above, has a multi-labeled distribution in the chest disease dataset. The Follow up column identifies X-ray data taken at different time intervals for the same patient. Patient ID contains unique identification numbers for each patient. Patient Age indicates patient ages, Patient Gender indicates patient genders, and View Position indicates X-ray imaging positions. These imaging positions are performed in two different ways: Postero-Anterior (PA) and Antero-Posterior (AP). PAs indicate standard exposures taken from the front of the patient, while APs indicate exposures taken with the patient in a lying position. In addition, the accompanying age factor [24] has a significant effect on patient exposures. Also, the heights and widths of the images, as well as the pixel values of the original image dimensions in the x and y coordinates, also play an important role in the dataset containing the clinical data.

The smaller dataset used in this study consists of 5606 images representing a pre-selected, random 5% sample of the full NIH Chest X-ray dataset available on Kaggle. The dataset was accessed via Kaggle as open-source data. The random distribution between classes was maintained by preserving the classes of the open-access dataset used in other studies in the literature. This subset was created through random sampling rather than patient-based stratification in order to reduce computational load while preserving the natural disease distribution hierarchy of the original data. This random sampling strategy was included in our study to facilitate the analysis of model performance in low-resource environments and to observe the effectiveness of the architecture on an external dataset.

**Figure 1:**
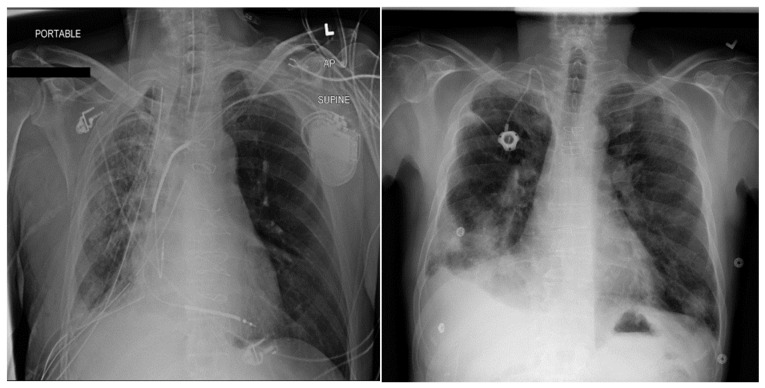
Sample Chest X-ray Images in the Dataset.

Figure 1 contains sample chest X-ray images from the dataset. The first image is an X-ray showing Emphysema, Infiltration, Pleural Thickening, and Pneumothorax. This X-ray image contains multiple pathologies simultaneously and is labeled as multi-labeled in the dataset. The second image contains Effusion and is treated as a single-labeled image in the dataset because it contains only one pathology.

The raw state of the dataset is as shown in the sample clinical data in Table 1 above and the sample X-ray data in Figure 1. Some preprocessing steps were performed to extract the dataset from its raw state and prepare it for model training. First, all diseases were subjected to one-hot encoding, and each disease was coded with 0 and 1. Images and data containing diseases were marked with 1, while those without diseases were marked with 0. Looking at recent studies [25], the one-hot encoding process works reliably on various datasets, but in order to achieve the best performance, a well-designed convolutional neural network (CNN) is generally required.

Table 2 shows the new data types after the one-hot encoding process of the dataset. Having all diseases in a single data type as int64 will facilitate the model’s learning process and improve its performance. The preprocessing steps performed so far and shown in the tables have been applied to the Random Sample of NIH Chest X-ray Dataset, and the same steps have been performed for the NIH Chest X-ray Dataset. The preprocessing steps applied do not differ structurally. After the data was converted to a single type, the distribution of diseases in the dataset was examined. The disease distribution for both datasets is shown in Table 3.

When examining the numerical distribution of the diseases listed in Table 3 across both datasets, it is observed that the distribution of diseases in the NIH Chest X-ray Dataset is relatively more consistent. Increasing the number of samples allows for better learning of disease representations, enabling the creation of more stable and generalizable models. On the other hand, although the Random Sample of NIH Chest X-ray Dataset preserves the distribution of diseases, it is thought that rare diseases are underrepresented due to the small sample size. This situation may cause an increase in variance in model learning. The No Finding label, present in both datasets, covers images and clinical data that do not contain pathological disease findings. This label does not directly resemble other disease labels in terms of pathological meaning. Consequently, only pathogenic classes were subjected to label-based distribution analysis, and the representation level of each disease in the dataset was appropriately evaluated.

### 2.2. Unimodal and Multimodal Models Preparation on the 5606-Sample Dataset

#### 2.2.1. Unimodal Models

In this study, unimodal models were constructed using chest X-ray image data. Unimodal models are simple and effective systems that process data of a single type, such as sound, face, or image. However, since they can only process a single type of data, they provide optimal results for specific and limited applications [26].

CNNs, which play an important role in detection and segmentation tasks, play a significant role in automating these functions. The ResNet50 architecture, an advanced framework of CNNs, is known for its deep residual learning structure. Its ability to manage the gradient problem while maintaining high accuracy values makes it a powerful and important tool for various image recognition tasks [27]. For this reason, in this study, the ResNet50 architecture, an advanced version of CNNs, will be trained for use in multi-label unimodal models using the Random Sample of NIH Chest X-ray Dataset, and its performance will be analyzed.

During the training process of the unimodal ResNet50 architecture, the dataset was initially divided into three parts: training, testing, and validation. For the final evaluation of the model’s performance, 20% of the dataset was designated as the test set. A second splitting procedure is applied to the remaining 80% of the data, with 20% allocated for validation and 80% for training. As a result, 20% of the dataset was used for testing, 16% for validation, and 64% for training. As a result of these splitting operations, the Random Sample of NIH Chest X-ray Dataset was configured as 3587 for the training set, 1122 for the test set, and 897 for the validation set.

The repeatability of the experiment was ensured by assigning a fixed randomness value to all partitioning operations of the dataset. The main objective here is to observe overfitting during the model training process and to ensure that the model’s performance can be evaluated objectively.

Since unimodal architectures will be built based on image data, the ImageDataGenerator structure of the Keras library was used to make the medical image data in the dataset suitable for the models. The pixel values of all chest X-ray images were scaled by a factor of 1/255 and normalized to the range [0, 1]. To meet the input requirements of the ResNet50 architecture, medical images were scaled to 224 × 224 pixels and processed in the RGB color space.

To feed the dataset into the model, separate data generators were created for the training, testing, and validation datasets. The file locations of medical images use a data frame to be matched with the corresponding multi-labeled disease data. During the training phase, the shuffling method was enabled within the data generator to prevent the model from learning spurious ordering patterns and to ensure robust generalization across epochs. Conversely, for the validation and testing phases, shuffling was explicitly disabled. This distinction ensures that the order of predictions strictly aligns with the ground-truth label vectors, facilitating the accurate calculation of label-based performance metrics such as AUROC and preventing data-label mismatch during the evaluation process.

To perform the image-based multi-label unimodal classification task, ResNet50, a pre-trained architecture on the ImageNet dataset, is used in the first stage. Using a pre-trained architecture instead of building a simple CNN architecture maximizes the feature extraction capability from images while leaving performance issues behind for the model to be built. The Transfer Learning process [28] performed in this way can transfer invariant information and features from one domain to another (from the source domain to the target domain) by examining the differences in error-related features and distributions between the two domains. In setting up this model, feature maps obtained from the last convolutional layer are converted into a one-dimensional feature vector using the global average pooling technique. This approach minimizes the risk of overfitting and reduces the number of model parameters by disabling the classification layers. The generated feature vector is transferred to the fully connected output layer, which is designed to match the number of diseases in the dataset. The sigmoid function [29], which is the activation function of the output layer, is a nonlinear component widely used in machine learning, and in shallow networks, it is commonly used in its derivative for error backpropagation and weight adjustment during both the training phase and the working phase (inference). For this reason, and also because a multi-label classification process is performed, the sigmoid function has been selected as the activation function in the output layer.

Within the training process of this architecture, various recall mechanisms are used to better monitor performance and clearly visualize model outputs. Based on the model’s validation loss, the model weights were saved at the point where the best performance was achieved during training. To achieve this, the ModelCheckPoint approach was used to save the weights at the training point where the model had the lowest validation loss.

The Adam algorithm was used in the model optimization phase, the learning rate was set to 1 × 10^−3^, and binary cross-entropy was chosen as the loss function. This allows each disease label to be evaluated independently. The ReduceLROnPlateau recall mechanism, which monitors validation loss, was used to dynamically change the learning rate throughout the training phase. To ensure the model converged more stably, the lower limit of the learning rate was set to 1 × 10^−8^, and if the validation loss did not improve within a predetermined period, the learning rate was set to decrease tenfold.

The single-mode ResNet50 architecture was trained using data generators containing image data. The model was trained for 20 epochs, and validation procedures were dynamically selected for each training cycle based on the size of the dataset. The number of samples in the relevant datasets was divided by the batch size to obtain the number of training and validation steps. The model’s performance was monitored at the end of each epoch, and the versions with the best weights were recorded using the determined recall functions. This prevented the risk of overfitting and ensured a balanced training by providing consistency and stability in the learning process. The loss function and AUROC (Area Under the Receiver Operating Characteristic) are used to evaluate the model’s performance. This method enables a more meaningful analysis of model performance, especially when there is class imbalance in multi-labeled medical datasets.

In this section, in addition to the ResNet50 architecture, the EfficientNetB3 and DenseNet121 architectures are also implemented to perform the multi-label single-mode classification task. Furthermore, these two developed models provide a comparative performance analysis aimed at performing the multi-label unimodal image-based classification task. By comparing CNN structures with varying depths, numbers of parameters, and feature extraction strategies under the same dataset and training strategy, the main objective of collectively analyzing these three architectures is to methodically examine the impact of architectural choices on multi-label chest disease classification.

Compared to more traditional CNNs, EfficientNet architectures are scaled composite models that provide a better balance between accuracy and computation [30]. EfficientNet, a family of highly efficient and scalable CNN architectures, has demonstrated state-of-the-art performance in various classification benchmark tests with fewer parameters and lower computational costs. EfficientNetB3, in particular, offers an excellent balance between model complexity and accuracy, making it ideal for use in real healthcare systems [31]. EfficientNetB3 was included in this study due to the balance it achieves between model complexity and accuracy. The main reason for choosing B3 among the EfficientNet architectures is that smaller variants may be insufficient in capturing pathological patterns, while larger variants may face the risk of overfitting. Therefore, by selecting a mid-level model, high computational costs were avoided, ensuring a more stable training process for clinical scenarios.

DenseNet is known for its interconnected and dense connection structure, which allows each layer to receive input from all previous layers. This facilitates the learning of complex patterns and the reuse of information with fewer parameters. Compact design and depth are beneficial when fine-grained features are crucial in medical imaging tasks [32]. The primary reason for selecting the DenseNet121 architecture for this research is its ability to process various pathological abnormalities and fine structural differences frequently observed in chest X-ray images, from early layers to higher levels. In multi-label classification tasks, the dense connection structure minimizes information loss across the network, enabling a more accurate representation of the unique features associated with each instance.

In order to evaluate the advantages of the three architectures discussed above within their own contexts and to perform a comprehensive comparative analysis, the preprocessing procedures specified for the training, validation, and testing partitioning strategy for the ResNet50 architecture were also applied identically to the EfficientNetB3 and DenseNet121 architectures, which were pre-trained on the ImageNet dataset. To solve the multi-label classification problem, these architectures also eliminate the upper layers of the basic network topologies and add a multi-output dense layer with a sigmoid activation function. The same training strategy, the same optimization technique, the same loss function (binary cross-entropy), and the same performance evaluation metrics were used to create a fair and uniform comparison environment for all models. Both models included were trained for 20 epochs. This method made it possible to accurately determine that performance differences stemmed from the representational power of the architectural structures rather than the training procedure or data processing stages. The figures below show the structure of unimodal models developed using the Random Sample of NIH Chest X-ray Dataset.

**Figure 2:**
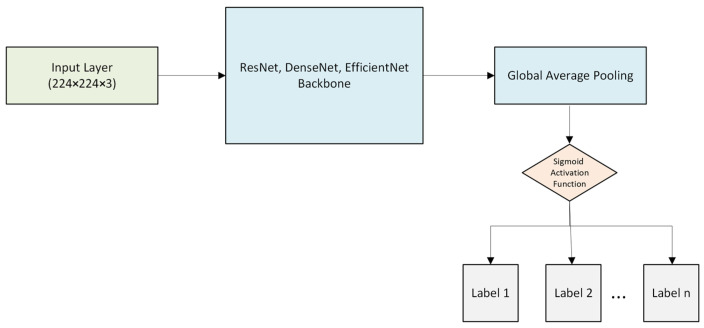
Multi-Label Unimodal Architectures.

Figure 2 below shows the block diagram of the multi-label unimodal EfficientNetB3 architecture. This model, which takes 224 × 224 × 3 image data as input, processes it in a series of steps (1–7) using MBConv blocks that maximize channel-based attention and convolutional efficiency. It uses a Global Average Pooling layer to vectorize feature maps in order to minimize dimensional complexity and prevent overfitting. To meet multi-label classification criteria, a fully connected layer with a sigmoid activation function produces independent label probabilities in the final step.

Figure 2 shows the block diagram of the unimodal ResNet50 architecture with multiple labels. This model uses the ResNet50 backbone in a hierarchical procedure to evaluate discriminative features. Based on deep residual learning concepts, this model uses residual bottleneck blocks instead of traditional convolutional stacks, reducing the vanishing gradient problem while ensuring a more accurate representation of spatial features through skip connections. Comprehensive labeling results from unimodal data are made possible by the independent neurons in the model’s final layer. The sigmoid function allows the probability of each class being present to be calculated simultaneously.

The unimodal multi-label DenseNet121 architecture shown in Figure 2 shares the same structure as EfficientNetB3 and ResNet50 regarding the output layers. Considered as another alternative approach for comparative analysis, this architecture functions as a feature extractor while incorporating densely connected layers. Unlike traditional CNN architectures, it maintains parameter efficiency and maximizes gradient flow by leveraging the unique internal structure of dense blocks, where each layer receives collective knowledge from all preceding layers.

#### 2.2.2. Multimodal Models

By analyzing and integrating various types of data using deep learning algorithms, multimodal deep learning—which combines multiple data types—has become a state-of-the-art method that enhances the accuracy and robustness of prediction models. Unlike traditional unimodal approaches, it uses various data sources, including genetic information, text data, and images, to provide more comprehensive information about complex biomedical events [33]. In recent years, multimodal systems that frequently utilize deep learning techniques have proven successful in biological applications. This facilitates more accurate and comprehensive analyses. Combining various data types under multimodal modalities achieves more accurate and comprehensive analyses as well as more accurate application results. In this regard, various fusion models used accomplish this by combining the features of various data types. The model’s generalization capacity is enhanced by the use of various data types [34].

In light of all this information, it is anticipated that the integration of image-based information along with features derived from clinical data in the relevant study will enable more accurate modeling of various disease anomalies. Within this framework, in addition to establishing unimodal models on the Random Sample of NIH Chest X-ray Dataset, multimodal deep learning strategies are also being applied.

In the first phase of the multimodal deep learning structure, the EfficientNetB3 architecture is preferred as the backbone to enable image data to be fed into the model. Clinical data undergoes appropriate preprocessing steps, after which the integration of image and clinical data is achieved within a fully connected network.

A traditional data splitting strategy is followed to preserve the sample matching between image data and clinical data. The dataset is divided into three subcategories by assigning a fixed randomness value: training, validation, and testing. In the first stage, 80% of the dataset is designated for training and 20% for testing. Then, the 80% portion is subjected to a second splitting process, with 20% allocated to validation and 80% to training. As a result, 3587 samples from the Random Sample of NIH Chest X-ray Dataset were categorized as training data, 897 as validation data, and 1122 as test data.

The clinical data in the dataset were separated to match the image data one-to-one. Three clinical features containing information about the patient’s gender and imaging position (AP/PA) (PatientGender_M, ViewPosition_AP, and ViewPosition_PA) were selected, converted to numerical form, and fed into the model. To make the clinical features compatible with deep learning models, they were created independently for each data section and converted to float32 data format. This was done so that the multimodal fusion strategy could perform the learning process without any information loss and capture consistent representation spaces.

All file paths for image data have been reorganized to enable immediate access to the relevant image file for each example. Images are loaded in RGB format, normalized to the [0, 1] range, and scaled to 224 × 224 pixels to match the model input size.

A unique multi-modal data generator structure derived from the Keras Sequence class has been created to ensure that two different modalities are fed into the model simultaneously. At each training stage, this structure generates image data and related clinical features in parallel. These parallel data groups are presented to the model together with label vectors in a suitable manner to enable multi-label classification. To prevent the model from falling into data sequence-related errors during training, the generator structure is configured to be shuffled during each training cycle. Inter-modality consistency in the multi-modal fusion strategy is thus ensured. Synchronized pairs were created for each image-clinical data batch structure, yielding vectors of dimensions (32, 224, 224, 3) for image inputs, (32, 3) for clinical inputs, and (32, 14) for multi-label outputs.

After all these data preparation steps are completed, a multi-labeled, multi-modal model is established using the Random Sample of NIH Chest X-ray Dataset. At this stage, a deep learning architecture based on EfficientNetB3, pre-trained on ImageNet, is developed to enable the joint evaluation and feeding of image and clinical data into the model. This model [35], preferred for representing image data, is a consistent and balanced structure in terms of computational efficiency and provides high performance, making it an ideal choice for clinical applications. This structure achieves high-level feature extraction and, as a result of the Global Average Pooling operation, reduces the spatial information density, obtaining a more compact and discriminative image representation.

This multimodal architecture, based on EfficientNetB3, incorporates clinical data as a three-dimensional vector. Processed through a single-layer, fully connected network topology, these clinical features are transferred to a nonlinear representation space. The goal here is to combine deep features extracted from images with clinical information in a more logical manner.

Information obtained from both modalities is combined through feature-level fusion. Feature-level fusion [36] combines features from various modalities into a high-dimensional representation. Thus, compared to previous layers, it enables intermodal interaction through more meaningful representations. Feature-level fusion was preferred to avoid disadvantages such as information inconsistency resulting from raw data merging in early fusion and the limited intermodality relationship in late fusion.

The sigmoid activation function preferred in the model’s output layer has a multi-labeled output layer. Therefore, separate prediction probabilities are obtained for each pathological case. Designed in this multi-modal way, the architecture supports image-based deep features with clinical context information to provide more robust and generalizable representation learning in the challenging task of multi-labeled chest disease classification.

To ensure fair comparability, the training configuration was standardized across models, with particular modifications made to meet the computational requirements of the large-scale dataset. With a minimum lower bound of 1 × 10^−8^, the optimization was carried out using the Adam optimizer with an initial learning rate of 1 × 10^−3^ that was dynamically decreased using the ReduceLROnPlateau scheduler upon validation loss stagnation. The large-scale dataset could only be trained for 10 epochs, whereas the smaller dataset could be trained for 20 epochs. Conversely, because of the large difference in dataset size, 10 epochs on the large dataset result in significantly more total gradient update steps than 20 epochs on the smaller subset, ensuring robust feature learning. This adjustment does not impede convergence. In order to balance convergence speed with GPU memory constraints, a fixed batch size of 32 was kept.

In multi-label classification tasks of this nature, the accuracy metric alone may be insufficient and may not provide adequate results for performance evaluation. For this reason, label-based AUROC values have been preferred in order to best evaluate the performance of this multi-label classification problem. This mechanism, which calculates the AUC value based on labels in the validation dataset at the end of each training cycle, enables more accurate measurement of the model’s discriminatory power, especially in medical datasets where class distributions are not equal. As the AUROC value on the validation dataset increases, the model weights are updated optimally in the relevant training cycle. As a result, when selecting a model, discriminative performance, which has greater clinical significance, is considered in addition to the loss value. This approach ensures a more unbiased evaluation of learning success, particularly for rare disorders. To ensure reproducibility during data splitting and model training, all trials were performed using a fixed randomization value throughout the training procedure. The goal of this training and evaluation approach is to obtain consistent, widely applicable, and clinically significant results from a multi-modal architecture.

To investigate the effect of various deep learning architectures on multimodal structures, ResNet50 and DenseNet121-based models were created in addition to the multi-labeled multimodal modeling procedure performed using the EfficientNetB3 architecture. The same data partitioning technique, clinical features, fusion approach, and training parameters were used. The aim here is to examine the changes in model performance that arise when only the image encoder architecture is changed. The ResNet50 and DenseNet121 architectures, initialized with pre-trained weights, integrated images and clinical data using a feature-level fusion strategy, as in EfficientNetB3. A fully connected layer was used to reflect the clinical features in the feature space. The resulting clinical and visual representation vectors were mixed and fed into a multi-label classification model. The training and evaluation protocols set for EfficientNetB3 were used in both developed architectures, and both architectures were trained for 20 epoch cycles. The goal of this approach is to ensure that the obtained performance results can be attributed to architectural differences. The figures below show the structure of the multimodal models developed using the Random Sample of NIH Chest X-ray Dataset.

**Figure 3:**
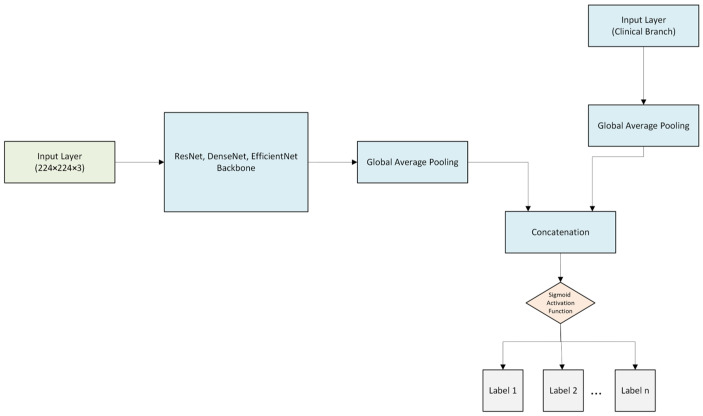
Multi-Label Multimodal Architectures.

Figure 3 below shows the schematic of the multi-label, multimodal EfficientNetB3 architecture. In this architecture, while feature extraction is performed from images using the EfficientNetB3 convolutional neural network, clinical data is processed in parallel using Dense layers. The concatenation layer combines the Global Average Pooling vector from the image branch and the 32-unit feature vector from the clinical branch into a single vector, enabling the model to learn from both input sources simultaneously. This combined feature map is fed into the Sigmoid activation function in the final stage to produce an output for multi-label classification.

The multi-label, multimodal ResNet50 architecture shown in Figure 3 has the same structure as the EfficientNetB3 architecture. As before, the Global Average Pooling layer functions for dimensionality reduction operations. The Dense layer, which processes clinical data with 32 neurons in a parallel branch, converts tabular data into feature space using the ReLU activation function. The preferred ReLU activation function [37] at this stage is one of the most frequently used and widespread methods in deep learning, preferred for its simplicity and computational efficiency. An output layer with a sigmoid activation function produces multi-labeled predictions in the final stage after the feature vectors from both branches are combined in the pooling layer to form an integrated data representation.

Looking at the multi-label, multimodal DenseNet121 architecture shown in Figure 3, it can be seen that it shares the same structure as the EfficientNetB3 and ResNet50 architectures. In the model, image data is processed using convolutional layers and Global Average Pooling. Global Average Pooling [38], found in almost every model, aggregates activations from the final convolutional layers and functions as learned representations. Clinical data is processed in a separate branch and fed into a nonlinear layer with 32 neurons. The image and clinical data branches, integrated in the concatenated layer, form an integrated representation that optimizes data correlation. At the final stage, a multi-label final classification prediction is produced with an output layer that incorporates a Sigmoid activation function.

To explicitly define the multimodal fusion framework, let I represent the input chest X-ray image and C denote the associated clinical metadata vector (comprising gender and view position). The visual feature representation, $f_{v}$, is derived by applying Global Average Pooling (GAP) to the feature maps generated by the final convolutional block of the backbone architecture, which minimizes spatial dimensions while retaining discriminative information. This extraction process is formulated as $f_{v}=\mathrm{GAP}\left(\Phi_{\mathrm{CNN}}\left(I\right)\right)$. Simultaneously, the clinical metadata C is projected into a compatible latent feature space $f_{c}$ through a fully connected dense layer with weight matrix $W_{c}$ and bias $b_{c}$, followed by a Rectified Linear Unit (ReLU) activation to introduce non-linearity, expressed as $f_{c}=\mathrm{ReLU}\left(W_{c}C+b_{c}\right)$. To integrate these heterogeneous modalities, we employ a feature-level concatenation strategy, where the normalized visual and clinical vectors are joined to form a unified representation $F_{\mathrm{fused}}=f_{v}⊕f_{c}$. This fused high-dimensional vector is subsequently fed into the final fully connected output layer with sigmoid activation σ to predict the probabilities $\hat{y}$ for the multi-label chest diseases, defined as $\hat{y}=\sigma \left(W_{out}F_{\mathrm{fused}}+b_{out}\right)$.

Although the NIH Chest X-ray dataset contains comprehensive metadata such as patient age, original image dimensions, and pixel spacing, this study limits the multimodal clinical input vector specifically to Patient Gender and Image Location. The exclusion of dimensional metadata except for OriginalImageWidth, OriginalImageHeight, and PixelSpacing is justified by the preprocessing pipeline, which rescales all input images to a uniform size of 224 × 224 pixels. This renders raw dimensional variances less critical for the CNN’s feature extraction process compared to standardized visual input. Consequently, the clinical domain focuses solely on the View Position and Gender variables due to their proven, significant effects on categorical structures and the morphological presentation of thoracic anatomy. This allows the model to learn specific modality interactions without the potential noise or redundancy introduced by continuous dimensional variables.

### 2.3. Unimodal and Multimodal Models Preparation on the NIH Chest X-Ray Dataset

#### 2.3.1. Unimodal Models

In this section, the impact of large-scale data on the performance of multi-label classification is investigated by creating unimodal deep learning models using the NIH Chest X-ray Dataset. In this design phase, the same model architectures and training processes will be used as in the Random Sample of NIH Chest X-ray Dataset. The goal is to fairly and rigorously compare the behavior of unimodal models under conditions of increased data availability.

Therefore, the preprocessing steps applied to the Random Sample of NIH Chest X-ray Dataset were applied in the same manner to the NIH Chest X-ray Dataset. This strategy ensures a fair comparison of model performance across various dataset scales and guarantees methodological consistency. In order to extract image descriptors and related diagnostic labels, the metadata file was parsed, and compound labels were split into parts and converted into a binary single-coded format to analyze multi-labeled disease descriptions. Labels containing the No Finding finding were cleaned to ensure that the learning process focused on pathological diseases. File path indexing was used to match medical chest X-ray images with relevant labels, ensuring the identification of valid image-label pairs.

Looking at the model development stage, the first unimodal architecture developed within the scope of the NIH Chest X-ray Dataset is EfficientNetB3. After performing the preprocessing steps mentioned above, which were also applied to the small-scale dataset, the dataset was divided into training, validation, and test subsets using a fixed randomness value to ensure data consistency. In the first stage, 20% of the data was separated for testing, and the remaining 80% was subjected to the resampling process. Thus, 64% of the NIH Chest X-ray Dataset were separated as training (71,756 examples), 16% as validation (17,940), and 20% as testing (22,424). This division allows for a comprehensive evaluation of performance while reducing the possibility of overfitting.

Image data has been downsampled to 224 × 224 pixels through a pixel-based rescaling process and then normalized. Each medical image data point can be associated with several pathological conditions using a multi-label learning technique. The EfficientNetB3 backbone, which uses pre-trained weights from ImageNet, was used in the architectural design phase. This enabled efficient feature extraction using transfer learning and accelerated convergence on the comprehensive medical dataset. Convolutional feature maps were independently predicted for each pathology’s disease probabilities using a fully connected layer containing a sigmoid activation function with the help of Global Average Pooling. The model was trained using the binary cross-entropy loss function and optimized using the Adam optimizer with an initial learning rate of 1 × 10^−8^.

To observe model performance, a unique AUROC-based recall mechanism was used in addition to monitoring loss values in the validation set. This allowed the AUC values for each disease class to be examined throughout each training cycle. A training plan was created over 20 epoch cycles, ensuring that the model’s weights were saved at the point where it achieved its highest values during this process. To ensure consistent and effective optimization, a learning rate scheduling methodology was also incorporated into this recall mechanism to dynamically decrease the learning rate when the validation loss plateaued.

The NIH Chest X-ray Dataset was subjected to multi-label unimodal chest X-ray classification using the EfficientNetB3 architecture, along with ResNet50 and DenseNet121 backbones. All preprocessing methodologies, data partitioning strategies, training setups, and optimization procedures were consistently maintained across all models for a methodical and fair comparison. The same training-validation-test split, learning rate schedule, loss function, and evaluation process were used to train each architecture for 20 epochs. ResNet50, which has become a classic backbone in deep learning strategies, was included in the comparative analysis on the full-scale dataset due to its superior performance, particularly in medical image segmentation tasks [39]. DenseNet121, which has densely connected layers enabling efficient gradient propagation and feedback of learned features between layers, was integrated into the training process of the original-sized dataset because it increased the model’s classification efficiency and reduced computational complexity [40]. Including these two different architectures with only a backbone change in the same preprocessing steps and training process not only maintains a consistent experimental design but also provides a controlled assessment of how various convolutional feature extractors affect classification performance on large-scale chest X-ray data.

The network designs shown earlier are shared by all unimodal architectures used in model designs containing the NIH Chest X-ray Dataset. The dataset size is the only variable that changes between experiments, and since the architectural elements remain the same, the relevant figures are reused as references.

#### 2.3.2. Multimodal Models

In the final subsection of this section, multi-labeled multimodal model setups will be performed on the NIH Chest X-ray Dataset. To maintain methodological consistency and fair comparison between model configurations, all preprocessing steps performed on unimodal data have been continued in the data preparation phase of multimodal models. To focus the learning challenge solely on diseased conditions, the No Finding label has been excluded from further analysis, consistent with previous experiments. Subsequently, each pathology has been represented as a binary indicator associated with its presence or absence in a given instance using a multi-labeled one-hot encoding approach.

When considering the model design phase, EfficientNetB3, a pre-trained architecture on ImageNet, was chosen for the image branch in the initial stage due to its advantageous balance between computational efficiency and representational capacity. This accelerated convergence and enabled the use of Transfer Learning protocols. In the architecture, the final convolutional feature maps are passed through a Global Average Pooling operation to extract high-level spatial information from each chest X-ray image and summarize it in a fixed-length feature vector. To process organized patient metadata, a clinical feature branch was created simultaneously. A three-dimensional clinical input vector containing the patient’s gender and imaging view position (AP and PA) is sent to this branch. The network learns a compact nonlinear representation of clinical information prior to fusion by passing clinical features through a fully connected layer with 32 neurons and a ReLU activation function.

For the full-scale original data size, intermediate fusion was preferred in the fusion strategy of EfficientNetB3, the first multi-label multimodal model recommended. Known as intermediate fusion or feature-level fusion [41], this strategy utilizes deep learning layers or feature extraction techniques. This fusion technique, which integrates separately learned modality-specific representations, was chosen because it was considered the most suitable fusion strategy within the scope of the relevant study. This fusion method enables the model to preserve its representational capacity by effectively capturing and integrating complementary information from both modalities. After feature fusion, disease probabilities for each target label were generated using a fully connected output layer incorporating a sigmoid activation function. Due to the multi-label nature of chest X-ray diagnosis, where multiple clinical diseases may coexist in a single image, the binary cross-entropy loss function was chosen as the optimization objective. As a result, the final model enables variable thresholding and clinically meaningful result interpretation by generating an independent probability estimate for each disease class.

The model architecture was initialized with a learning rate of 1 × 10^−8^ using the Adam optimizer algorithm, and the learning rate was dynamically reduced using the ReduceLROnPlateau technique when the validation loss plateaued. This ensures training stability and minimizes the risk of overfitting. Considering the size and complexity of the dataset, the batch size was set to 32, and the training cycle was set to 10 epochs. This ensured consistency and balance between convergence speed and GPU memory constraints. In the performance evaluation phase, in addition to monitoring loss values, a special AUROC system calculated for all pathological cases at the end of each training cycle was used. A special call mechanism was created to record the weights at the training point where the highest AUC value was obtained. This approach not only optimizes to minimize loss but also ensures that the selected model maximizes its discriminatory performance across all disease categories.

In addition to the multi-label, multimodal EfficientNetB3 architecture developed using the NIH Chest X-ray Dataset within the scope of this study, two different convolutional backbone networks, ResNet50 and DenseNet121, are also used to examine the effect of backbone selection on multi-label, multimodal chest disease classification performance in greater detail. These architectures were selected because of their unique feature extraction paradigms and their widespread use in the medical imaging literature, as with the other models developed in this study. To ensure a fair and controlled comparison, all elements of the multimodal learning pipeline, except for the image feature extractor backbone, were kept constant across the developed models. Specifically, the preprocessing procedures, dataset splits, multimodal data generators, clinical feature encoding, fusion approach, loss function, optimization process, recall mechanisms, and training schedule of the multimodal EfficientNetB3 configuration were handled in the same manner. As with EfficientNetB3, the ResNet50 and DenseNet121 architectures were also trained over a 10-epoch training cycle, and their performance was observed. To ensure consistency in performance evaluation in the developed ResNet50 and DenseNet121 architectures, the best-performing model checkpoints were selected using the same AUROC callback, and the weights were recorded.

Using this standardized experimental protocol, the observed performance differences between the multi-label, multimodal EfficientNetB3, ResNet50, and DenseNet121 models can be attributed primarily to the unique representational properties of their backbone architectures, rather than differences in training strategy or data processing. The aim is to provide insight into how architectural decisions affect scaled cross-modal feature integration and to enable a logical assessment of backbone effectiveness in the context of multimodal medical imaging.

A common architectural structure for all multi-labeled multimodal architectures used in this study is represented by the multi-labeled multimodal network designs shown in the Figure 3. The same architectural configurations were used in multimodal studies conducted using the NIH Chest X-ray Dataset; the only difference between experiments was the dataset size. Since the architectural features were preserved in terms of image and clinical data processing, fusion method, and classification layers, the relevant figures were quoted and used as representations of the architectural structure.

## 3. Results

### 3.1. Results of Unimodal and Multimodal Models in the 5.606 Dataset

#### 3.1.1. Unimodal Models

In this section, the performance of multi-label unimodal deep learning models trained on the Random Sample of NIH Chest X-ray Dataset will be analyzed. For this purpose, the AUROCs calculated according to the disease for the EfficientNetB3, ResNet50, and DenseNet121 architectures, which only accept medical image data as input, are presented in this section to examine their classification performance in more detail.

**Figure 4:**
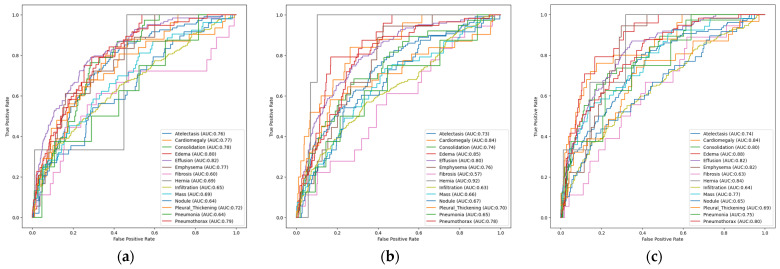
Class-Based ROC Curve of the Multi-Label Unimodal Architectures (**a**) EfficientNetB3, (**b**) ResNet50, (**c**) DenseNet121.

Figure 4a shows the disease-based ROC curves and AUC values of the unimodal, multi-label EfficientNetB3 architecture trained on the Random Sample of NIH Chest X-ray Dataset. The ROC curves and AUC values calculated independently for each pathology were used to evaluate the model’s classification performance. The model’s excellent discriminatory power is demonstrated by the high AUC values achieved, particularly in the Effusion (AUC: 0.82), Edema (AUC: 0.80), and Pneumothorax (AUC: 0.79) classes. These findings demonstrate that the model can accurately identify and distinguish the distinct morphological changes in radiological images. However, the modest size of these abnormalities in radiological imaging or their visual similarity to other classes may explain the relatively low performance seen in classes such as Fibrosis (AUC: 0.60) and Nodule (AUC: 0.64). Nevertheless, the fact that the AUC values of most classes in the EfficientNetB3 architecture are above the 0.70 threshold proves that this architecture demonstrates consistent and balanced performance in multi-label medical diagnosis problems. In other words, the model is statistically successful in distinguishing complex radiological data.

Figure 4b presents AUROCs, providing a comprehensive analysis of the AUC values based on pathological cases for the unimodal ResNet50 architecture with multi-label classification. As indicated by the ROC curve, the model has demonstrated exceptional performance, particularly in the Hernia (AUC: 0.92), Edema (AUC: 0.85), and Cardiomegaly (AUC: 0.84) classes. AUC values greater than 0.90 indicate that the model has a high degree of clinical reliability in distinguishing the relevant pathology from healthy tissue. When the overall performance of the model is examined, it is seen that the vast majority of classes achieve AUC values of 0.75 and above. The fact that similar results are achieved in important findings such as Effusion (AUC: 0.80) and Pneumothorax (AUC: 0.78) demonstrates how well the model detects anomalies of vital importance in radiological scans. However, the relatively weak performance of the Fibrosis (AUC: 0.57) and Infiltration (AUC: 0.63) classes can be explained by their finer radiological features and the small sample size of the dataset.

Figure 4c shows the ROC curve, where the AUC values calculated for each pathology indicate that the architecture demonstrates high-level discrimination and performance, particularly in Edema (AUC: 0.88), Hernia (AUC: 0.84), Cardiomegaly (AUC: 0.84), Effusion (AUC: 0.82), and Emphysema (AUC: 0.82). This demonstrates that the model exhibits high clinical reliability, particularly for these diseases. However, when looking at the pathologies of Fibrosis (AUC: 0.63), Infiltration (AUC: 0.64), and Nodule (AUC: 0.65), which are relatively lower than the others, it is possible to explain these low scores with lesion visual complexity and low contrast features. Nevertheless, when examining the overall performance of the DenseNet121 architecture, it is clear that it demonstrates strong performance in multi-label classification problems and achieves balanced ROC curves for the vast majority of classes.

#### 3.1.2. Multimodal Models

In this section, the performance results of multi-label, multimodal deep learning models trained on the Random Sample of NIH Chest X-ray Dataset will be evaluated. To provide a comparative analysis of the learning process and the effect of multimodal data integration on discriminative performance using ResNet50, EfficientNetB3, and DenseNet121 architectures, class-based ROC curves calculated to examine the loss values and classification performance associated with the training process of the models in detail are provided.

**Figure 5:**
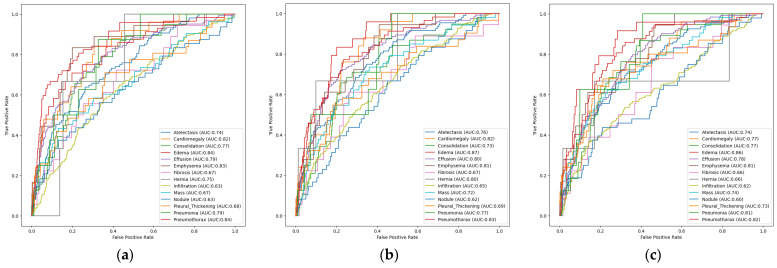
Class-Based ROC Curve of the Multi-Label Multimodal Architectures (**a**) EfficientNetB3, (**b**) ResNet50, (**c**) DenseNet121.

When examining the pathology-based ROC curve and AUC values of the multimodal EfficientNetB3 architecture with multiple labels, as shown in Figure 5a, it is observed that multimodal fusion increases modality accuracy and consistency. According to the analysis results, the model demonstrated strong discriminatory power in the Pneumothorax (AUC: 0.84), Edema (AUC: 0.84), Emphysema (AUC: 0.83), and Cardiomegaly (AUC: 0.82) classes. It showed consistent performance in important diagnostic classes such as Effusion (AUC: 0.79) and Pneumonia (AUC: 0.79), demonstrating that its effectiveness is not limited to specific diseases. However, it relatively lags behind in pathologies such as Nodule (AUC: 0.63), Infiltration (AUC: 0.63), and Mass (AUC: 0.67). Nevertheless, the balanced ROC curves and high AUC values of the multi-label, multimodal EfficientNetB3 architecture provide higher clinical reliability in multi-label medical diagnostic tasks compared to single-modal modalities.

According to the class-based ROC curve analysis of the multi-labeled, multimodal ResNet50 model shown in Figure 5b, the best results were obtained for Edema (AUC: 0.87), Pneumothorax (AUC: 0.83), Cardiomegaly (AUC: 0.82), Emphysema (AUC: 0.81), and Hernia (AUC: 0.80) pathologies. This indicates a high level of discrimination ability, particularly for these diseases. Overall, as can be seen from the clustering of the class curves in the upper left corner, this model, with its excellent sensitivity and low false positive rate, provides a strong foundation for medical decision support procedures.

Figure 5c shows the pathology-based AUC values of the multi-label, multimodal DenseNet121 architecture under the ROC curve. The model demonstrates high AUC scores in the Edema (AUC: 0.86), Pneumothorax (AUC: 0.82), Emphysema (AUC: 0.81), and Pneumonia (AUC: 0.81) classes, proving its clinical reliability. Overall, the model provides high sensitivity with a low false positive rate, as seen by the rapid increase in the curves towards the upper left corner in most classes. These findings demonstrate that the architecture effectively combines medical images and clinical data to address the challenge of multi-labeled medical classification.

### 3.2. Results of Unimodal and Multimodal Models in the NIH Chest X-Ray Dataset

#### 3.2.1. Unimodal Models

**Figure 6:**
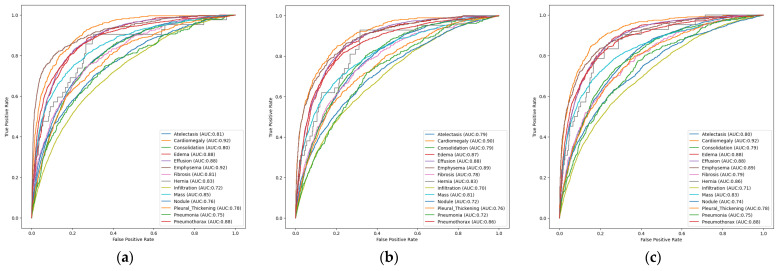
Class-Based ROC Curve of the Multi-Label Unimodal Architecture (**a**) EfficientNetB3, (**b**) ResNet50, (**c**) DenseNet121.

In this section, the loss values and AUROCs of three different unimodal multi-label architectures designed using the NIH Chest X-ray Dataset will be analyzed. Based on the class-based ROC curve of the multi-labeled unimodal EfficientNetB3 architecture in Figure 6a, it has demonstrated exceptional performance, particularly in the Cardiomegaly (AUC: 0.92) and Emphysema (AUC: 0.92) classes. The fact that pathologies such as Edema (AUC: 0.88), Effusion (AUC: 0.88), and Pneumothorax (AUC: 0.88) also exceed the 0.80 threshold proves that the model recovers very strong features from the large dataset. The lowest score was obtained for Infiltration (AUC: 0.72). The fact that all diseases generally exceeded the 0.70 threshold and achieved superior performance maximizes the model’s generalization capacity for each disease as the amount of data increases.

Figure 6b examines the pathology-based AUC values of the multi-labeled unimodal ResNet50 architecture under the ROC curve. Cardiomegaly (AUC: 0.90), Emphysema (AUC: 0.89), Edema (AUC: 0.87), and Pneumothorax (AUC: 0.86) pathologies demonstrate superior performance, highlighting the architecture’s pathological discrimination power. The lowest score in the figure was obtained for the Infiltration pathology (AUC: 0.70). However, overall, the fact that all pathologies have high AUC scores indicates that the architecture can learn complex radiological patterns accurately and successfully.

Figure 6c shows the ROC curve examining the pathology-based AUC values of the multi-label unimodal DenseNet121 architecture. This model, which achieves high AUC scores in cardiomegaly (AUC: 0.92), emphysema (AUC: 0.89), and pneumothorax (AUC: 0.88) pathologies, demonstrates how large-scale data maximizes classification success in deep structures. The model’s ability to learn and generalize visual differences has reached clinical-level reliability, as seen in the momentum gathered in hard-to-detect classes. Accordingly, the high discrimination power and low false positive rate that the NIH Chest X-ray Dataset adds to the DenseNet121 design are confirmed by the steady trend of all class curves towards the upper left corner.

#### 3.2.2. Multimodal Models

**Figure 7:**
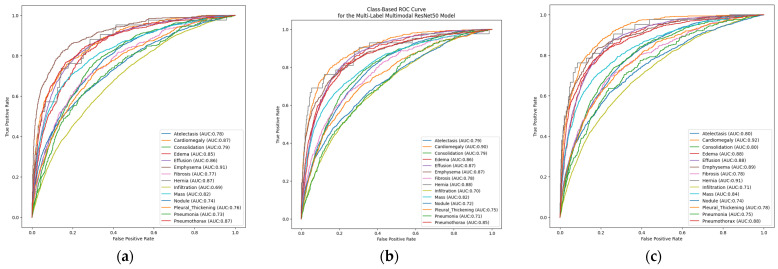
Class-Based ROC Curve of the Multi-Label Multimodal Architectures (**a**) EfficientNetB3, (**b**) ResNet50, (**c**) DenseNet121.

In this subsection, the loss values and ROC curves of multi-label multimodal architectural designs trained using the NIH Chest X-ray Dataset will be analyzed. Figure 7a examines the class-based AUC values under the ROC curve of the multi-label, multimodal EfficientNetB3 architecture. This figure clearly shows the model’s overall effectiveness in distinguishing various pathologies and the performance difference between classes. The model demonstrated excellent discriminatory power and clinically significant performance, particularly in classes such as Emphysema (AUC: 0.91), Pneumothorax (AUC: 0.87), and Cardiomegaly (AUC: 0.87). Although relatively low performance was observed in classes such as Infiltration (AUC: 0.69), the curves for all classes are above the diagonal line. These low scores indicate problems arising from visual similarities in the data. Overall, the observed AUC values indicate that the model can compete with similar studies in the literature and provides good reliability in multi-label classification tasks after being trained on the NIH Chest X-ray Dataset.

Figure 7b shows the ROC curve of the multi-label, multimodal ResNet50 model. The ROC analysis of the ResNet50-based multimodal model shows that the system performs exceptionally well and has a high success rate in distinguishing abnormal signals, particularly in the Cardiomegaly (AUC: 0.90) and Hernia (AUC: 0.88) classes. The model’s strong prediction ability on the NIH Chest X-ray Dataset is evidenced by the fact that the AUC values of most classes are above or close to the 0.80 threshold.

Although the model’s clinical diagnostic ability is relatively lower in pathologies such as Infiltration (AUC: 0.70), it is evidenced by the fact that all curves generally tend towards the upper left corner of the figure with high scores. The findings obtained from the figure show that this architecture can be applied as a reliable classifier in multi-labeled medical imaging tasks.

Figure 7c presents the AUROCs for the multi-labeled, multimodal DenseNet121 architecture. This figure indicates that the conditions of Cardiomegaly (AUC: 0.92) and Hernia (AUC: 0.91) exhibit clinically significant discriminative performance, surpassing all other categories. As seen in the figure, the fact that most pathologies have an AUC score above the 0.80 threshold proves the model’s generalization ability. This highlights the suitability of the relevant architecture for clinical decision support mechanisms. Even in classes with low scores on the curve, such as Infiltration (AUC: 0.71), a good starting point was provided, showing that the benefits of deep feature reuse are effectively preserved in a multimodal framework. The overall findings confirm that DenseNet121 provides a largely balanced and reliable performance in radiological finding classification.

## 4. Discussion

This study systematically investigated multi-labeled unimodal and multimodal deep learning methods across different data scales for the classification task of 14 distinct chest diseases. The results enable an assessment of the impact of data volume on model performance, alongside the contributions of multimodal integration. The study used two different versions of the NIH Chest X-Ray dataset: the Random Sample of NIH Chest X-ray Dataset, consisting of 5606 samples, and the NIH Chest X-ray Dataset, consisting of 121,120 samples. The unimodal models developed in the study only included medical image chest X-ray data, while the multimodal models provided clinical data and medical image integration. A total of 12 models were developed throughout the study. Among these models, three basic advanced CNN architectures, namely ResNet50, EfficientNetB3, and DenseNet121, were used when handling image data. The 12 different models shaped by these three basic architectures demonstrate how different dataset sizes affect unimodal and multimodal models. According to the results obtained from the Random Sample of NIH Chest X-ray Dataset, unimodal models have come to the fore in some diseases but have generally lagged behind multimodal fusion models. The small amount of data and the low representativeness of clinical variables are the most important factors affecting the performance of the models. Therefore, multimodal fusion, achieved through adequate and balanced data representation and appropriate architectures, has outperformed unimodal modalities.

We evaluated the performance of our models primarily using label-based AUC metrics, which is the standard approach for the NIH Chest X-ray dataset due to its multi-label nature. Since a single radiographic image can simultaneously exhibit findings for multiple pathologies (e.g., Mass and Pneumonia together), the classes are not mutually exclusive. Consequently, generating a traditional confusion matrix would require applying a fixed acceptance threshold (e.g., 50%) across all labels, which may not capture the true discriminative power of the model for rare or complex diseases. To avoid the methodological limitations and potential bias introduced by arbitrary thresholding in a multi-label setting, we focused on class-wise ROC curves and AUC scores, which provide a threshold-independent and more robust assessment of model reliability and class imbalance handling consistent with the prevailing literature.

**Figure 8:**
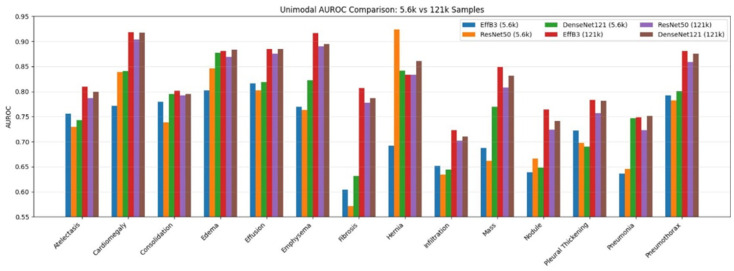
Comparison of Unimodal Per-Label AUC Scores at Sample Sizes of 5.6 k vs. 121 k.

Figure 8 shows a comparison of disease-based AUC scores for unimodal architectures designed using datasets of different sample sizes (5.6 k and 121 k). As can be seen from the figure, overall performance consistently improves across all disease labels when the data size is increased from 5.6 k to 121 k. This highlights the critical importance of data scale in clinical applications. Models trained on 121 k data demonstrated superior performance to those trained on 5.6 k data in almost all pathologies. There are particularly noticeable improvements in conditions with subtle and delicate visual differences, such as Fibrosis, Mass, Nodule, and Infiltration. In some cases, these classes show an AUROC gain of more than 0.10. This indicates that greater data accessibility provides greater robustness and generalization.

When examined in terms of architectures, EfficientNetB3 and DenseNet121 architectures have demonstrated better performance on large-scale datasets. They have once again proven their robustness by achieving AUC values above 0.90, particularly in pathologies such as Edema, Emphysema, and Cardiomegaly. On the other hand, performance differences between architectures are more irregular in small-scale contexts, indicating that data scarcity increases architectural sensitivity and limits the models’ ability to obtain discriminative representations. The fact that high scores are achieved even in low-data regimes, particularly for pathologies such as Hernia, indicates that these conditions exhibit more characteristic radiographic patterns. On the other hand, classifications such as Fibrosis and Infiltration remain difficult to detect with small amounts of data, highlighting the need for large-scale datasets to accurately identify subtle pathological features.

Overall, the findings indicate that in unimodal data fusion, the size of the dataset has a greater impact on classification performance than the choice of architecture. The use of large-scale data clearly enables CNN architectures to achieve clinically significant levels of discrimination in various chest diseases.

**Figure 9:**
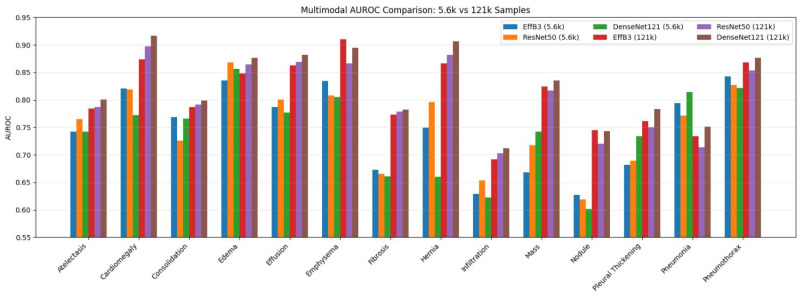
Comparison of Multimodal Per-Label AUC Scores at Sample Sizes of 5.6 k vs. 121 k.

Figure 9 shows how different dataset sizes in multimodal data fusion affect disease-based AUC scores across different architectures. As evident from the figure, multi-modal data fusion generally improves classification performance compared to single-modal fusion, particularly in diseases where inter-class similarity is high and visual signals are subtle. Models trained on the 121 k dataset show significant and consistent performance improvements across all disease classes, very similar to unimodal results. However, improvements and generalization capabilities are more pronounced in the multimodal fusion framework. This indicates that additional data inputs, such as clinical data integration, enable more efficient utilization of large-scale data. Significant AUROC gains are observed when looking at pathologies such as Mass, Nodule, Infiltration, and Fibrosis. In other words, this highlights the capacity of multimodal representations to capture complementary clinical and radiological information.

When evaluated based on architectures, DenseNet121 and EfficientNetB3 architectures generally achieved more stable and higher AUC scores than ResNet50. This is particularly evident in pathologies such as Cardiomegaly, Emphysema, Hernia, and Pneumothorax, where the 0.90 threshold was clearly exceeded within this framework. As can be seen from the results obtained from this figure, multimodal deep learning fusion confirms its clinical importance in the complex task of thoracic disease classification and detection. Multimodal deep learning fusion not only increases classification accuracy but also specifically enhances robustness and consistency across pathologies in large-scale data regimes.

In contrast, findings from the NIH Chest X-ray Dataset reveal the impact of both increased data volume and multimodal fusion on architectures. Architectural performance showed more consistent and stable improvement. Training medical image data together with clinical data enabled better representation of pathological overlaps within the scope of multi-labeled classification. Multimodal fusion strategies, in particular, demonstrated greater effectiveness with the increase in data size, once again outperforming single-modal modalities.

Within the scope of this study, three different basic CNN backbones were used: EfficientNetB3, ResNet50, and DenseNet121. These architectures demonstrated superior performance in both multimodal fusion and large-scale datasets. All architectures were developed using the same preprocessing steps, training protocols, and recall mechanisms to enable a fair and systematic comparison.

Despite all the advantages mentioned above, the study in question also has some limitations. For example, the components of the clinical data in the dataset included in the study are limited. The absence of additional information, such as laboratory values, time series, and doctor reports for patients, complicates the task of multimodal fusion and prevents it from fully demonstrating its performance. Class imbalances caused by rare diseases in the dataset can affect both single-modal and multimodal data fusion, reducing their performance. Considering this situation, even if a large-scale dataset is used, it should not be forgotten that there are still unbalanced classes in the dataset and that this situation affects model learning. Although two different versions of the dataset were used in the study, the data were obtained from a single data source. This leads to the models performing in a manner dependent on a single data source. Since there is no information obtained from different data sources or clinical centers, the generalizability of the models is limited.

The input vector of the multimodal model has been reduced to a standard 224 × 224 pixel size during the preprocessing stage. The clinical module focuses solely on the variables of gender and imaging position, which directly affect the morphological presentation and categorical structure of thoracic anatomy. In the context of the integration strategy, fusion at the feature level has been adopted, where visual and clinical representations are combined in a latent space. This approach enables the model to learn complementary relationships between heterogeneous sources more effectively by combining high-dimensional representations obtained from different data types into a coherent vector.

Table 4 shares the average AUC and standard deviation values of different fusion strategies for unimodal and multimodal approaches. The effectiveness of the Feature-Level Fusion strategy proposed in our study is also compared with the Late Fusion strategy in Table 1. Upon examining the table, it is observed that the unimodal image-based approach exhibits relatively low performance (mean AUC = 0.4750) and has limited discriminative power compared to multimodal methods. Multimodal late fusion strategies provide a significant improvement over the unimodal approach; however, performance differences between different weighting and fusion methods are limited, and the highest success is achieved with maximum fusion (mean AUC = 0.5693). In contrast, it is noteworthy that feature-level fusion methods offer significantly higher performance than late fusion in all configurations. In particular, the maximum fusion strategy achieving the highest average AUC value (0.7447) demonstrates that combining features obtained from different modalities at an early stage significantly increases the model’s discriminative power. The fact that the standard deviation values generally remain within similar ranges supports the stability of the results and confirms that the performance increase is not random. Furthermore, the results reveal that the Feature-Level Fusion technique yields more successful results compared to the Late Fusion strategy.

Within the proposed multimodal fusion framework, clinical variables are not analyzed as independent scalar predictors but are instead encoded into a consistent dense vector to represent the clinical acquisition context. This approach of the proposed model addresses the limited dimensionality issue of existing metadata compared to complex visual feature maps. This method, used within our architecture, enables the classification model to capture the interaction between patient demographics and image morphology rather than isolated variable effects. Therefore, the observed performance difference between unimodal and multimodal baselines serves as a holistic ablation study that directly measures the contribution of the clinical metadata layer to diagnostic accuracy. The differences between unimodal and multimodal approaches serve as ablation differences where architectural differences are examined.

The use of low-dimensional clinical vectors creates a fundamental distinction between demographic calibration and true semantic synergy. The use of variables such as gender and viewpoint position within the multi-model functions as conditional connection points that adjust probability distributions based on known epidemiological prevalence, rather than resolving visual ambiguities through complex feature alignment. Unlike models that use dense narrative data to fill semantic gaps, our feature-level fusion strategy was conducted within the scope of our study. This optimizes performance by reducing epistemic uncertainty regarding patient demographics.

The random sample subset consisting of 5606 images used in the analyses was created through global random sampling rather than patient-level splitting in order to reduce the computational load and lay the groundwork for similar resource-constrained studies in the literature. Given that patient-level splitting in datasets of this scale could lead to serious class imbalance by causing rare pathologies to be absent from either the training or test sets, preserving class distribution was prioritized, even at the risk of data leakage. However, the performance evaluations presented in the study are based on the performance improvement on the full NIH dataset of 121,120 samples, where the data leakage effect is statistically dampened within the large data volume and the model learns a wider variation, rather than directly on this small subset. Therefore, the small dataset serves as a reference point for analyzing the model’s behavior under limited data conditions and demonstrating the stability provided by large-scale data integration.

In the multi-label classification of chest diseases, the simultaneous presence of multiple pathologies in a single radiographic image and the heterogeneity of distribution across classes limit the use of metrics based on fixed threshold values in evaluating model performance. In this study, setting an arbitrary decision threshold of 50% would indeed carry the risk of masking the model’s discrimination capacity, particularly in rare diseases. Therefore, the performance analyses of the proposed architectures are built on label-based AUROC dynamics, a more robust metric independent of the threshold value. Rather than a direct comparison of numerical results, the systematic effect of increased data volume and multi-modality integration on diagnostic accuracy has been demonstrated in the conducted research.

Analysis of performance by class reveals that multimodal fusion provides diminishing returns for pathologies characterized by fine, localized overlapping opacities, such as Infiltration and Nodule. The current fusion framework relies on the direct combination of visual and clinical feature vectors rather than attention-based gatekeeping mechanisms. The proposed model handles clinical variables and visual features with fixed integration weights. While this approach is quite effective for conditions with strong demographic correlations, such as cardiomegaly, it struggles to resolve diagnostic uncertainty when the visual signal is weak and the available clinical metadata lacks specific discriminatory power for the pathology in question.

## 5. Conclusions

This study presented a systematic and large-scale comparative evaluation of unimodal and multimodal deep learning approaches for multi-label chest disease classification using chest X-ray images and associated clinical metadata. By employing three widely adopted convolutional neural network architectures—ResNet50, EfficientNetB3, and DenseNet121—across two distinct data regimes, the study comprehensively investigated the effects of modality integration and dataset scale on diagnostic performance.

The experimental findings consistently demonstrate that multimodal models integrating image and clinical information outperform their unimodal counterparts under both small- and large-scale data conditions. In particular, multimodal fusion yielded higher and more stable label-based AUROC values across a wide range of chest pathologies, indicating improved discriminative capability and robustness. These gains became more pronounced as the dataset size increased, highlighting the complementary role of data scale and modality diversity in enhancing model generalization. While unimodal models benefited from increased data volume, their performance improvements remained limited compared to multimodal approaches, especially for complex or visually subtle pathologies.

Moreover, the results confirm that maintaining a consistent architectural and training framework across different data scales enables a fair assessment of model behavior and reveals the true impact of modality integration. The use of multi-label learning further reflects realistic clinical scenarios, where multiple thoracic conditions may coexist within a single patient, thereby increasing the clinical relevance of the proposed framework.

Overall, this study provides strong evidence that multi-label multimodal deep learning constitutes a reliable and scalable strategy for chest disease diagnosis. The findings support the adoption of multimodal decision support systems in clinical practice, particularly in radiology workflows requiring robust interpretation of heterogeneous data sources. Future research directions include the exploration of advanced fusion strategies, attention-based and transformer-driven architectures, and the incorporation of richer clinical and temporal data to further enhance interpretability, semantic coherence, and diagnostic accuracy in multimodal medical imaging systems.

## Figures and Tables

**Table 1:** Sample Clinical Data in the Dataset.

Columns of Clinical Data	Sample Data
Image Index	00000013_005.png
Finding Labels	Emphysema|Infiltration|Pleural_Thickening|Pneumothorax
Follow up#	005
Patient ID	00000013
Patient Age	060Y
Patient Gender	M
View Position	AP
OriginalImageWidth	3056
OriginalImageHeight	0.139
OriginalImagePixelSpacing_x	2544
OriginalImagePixelSpacing_y	0.139

**Table 2:** Data Types After One-Hot Encoding of the Dataset.

Column	Non-Null Count	Dtype
Image Index	5606 non-null	Object
Finding Labels	5606 non-null	Object
Follow-up	5606 non-null	Int64
Patient ID	5606 non-null	Int64
PatientAge	5606 non-null	Int64
OriginalImageWidth	5606 non-null	Int64
OriginalImageHeight	5606 non-null	Int64
OriginalImagePixelSpacing_x	5606 non-null	Float64
OriginalImagePixelSpacing_y	5606 non-null	Float64
Patient Gender_M	5606 non-null	Bool
View Position_AP	5606 non-null	Bool
View Position_PA	5606 non-null	Bool
Atelectasis	5606 non-null	Int64
Cardiomegaly	5606 non-null	Int64
Edema	5606 non-null	Int64
Effusion	5606 non-null	Int64
Emphysema	5606 non-null	Int64
Fibrosis	5606 non-null	Int64
Hernia	5606 non-null	Int64
Infiltration	5606 non-null	Int64
…	…	…
Pneumonia	5606 non-null	Int64
Pneumothorax	5606 non-null	Int64

**Table 3:** Disease Distributions in Datasets.

Disease Labels	Random Sample of NIH Chest X-Ray	NIH Chest X-Ray 14
No Finding	3044	60,361
Infiltration	967	19,894
Effusion	644	13,317
Atelectasis	508	11,559
Nodule	313	6331
Mass	284	5782
Pneumothorax	271	5302
Consolidation	226	4667
Pleural_Thickening	176	3385
Cardiomegaly	141	2776
Emphysema	127	2516
Edema	118	2303
Fibrosis	84	1686
Pneumonia	62	1431
Hernia	13	227

**Table 4:** Average AUC Values for Different Fusion Strategies for Unimodal and Multimodal Approaches.

Modality	Fusion Strategy	Rate	Mean AUC	Standard Deviation
Unimodal Image	-	-	0.4750	0.0910
Multimodal	Late Fusion	(0.50–0.50)	0.5624	0.0801
Multimodal	Late Fusion	(0.40–0.60)	0.5600	0.0798
Multimodal	Late Fusion	(Max Fusion)	0.5693	0.0887
Multimodal	Late Fusion	(Min Fusion)	0.4930	0.0954
Multimodal	Feature-Level Fusion	(0.50–0.50)	0.7106	0.0814
Multimodal	Feature-Level Fusion	(0.40–0.60)	0.7144	0.0751
Multimodal	Feature-Level Fusion	(Max Fusion)	0.7447	0.0813
Multimodal	Feature-Level Fusion	(Min Fusion)	0.7273	0.0883

## Data Availability

The data presented in this study are openly available in [Chest X-Ray dataset] at [https://nihcc.app.box.com/v/ChestXray-NIHCC accessed on 1 July 2025].
